# Supplementary material for: Epidemiology and clinical profile of diarrhea associated with enterotoxigenic Escherichia coli and Vibrio cholerae in Bangladesh: Findings from a hospital-based surveillance system, 2008–2023
Source: PLoS Negl Trop Dis. 2025 Nov 7;19(11):e0013697. doi: 10.1371/journal.pntd.0013697 (PMC12611153; doi:10.1371/journal.pntd.0013697)
Supplement: S1 Table — (DOCX) [file pntd.0013697.s001.docx]

**S1 Table:** Clinical features according to age group for pathogen

| **Variables** | ***V. cholerae*** | | | | **ETEC** | | | | **Co-infection** | | | |
| --- | --- | --- | --- | --- | --- | --- | --- | --- | --- | --- | --- | --- |
|  | **Age**  **<5 years**  **729 (%)** | **Age**  **5-<15 years**  **546 (%)** | **Age**  **15-<60 years**  **3044 (%)** | **Age**  **>60 years**  **220 (%)** | **Age**  **<5 years**  **1539 (%)** | **Age**  **5-<15 years**  **84 (%)** | **Age**  **15-<60 years**  **1270 (%)** | **Age**  **>60 years**  **184 (%)** | **Age**  **<5 years**  **115 (%)** | **Age**  **5-<15 years**  **42 (%)** | **Age**  **15-<60 years**  **437 (%)** | **Age**  **>60 years**  **36 (%)** |
| **Duration of diarrhea before arrival at hospital** | | | | |  |  |  |  |  |  |  |  |
| <1 day | 341 (46.8) | 374 (68.5) | 2239 (73.5) | 170 (77.3) | 570 (37.3) | 58 (69.1) | 825 (65.0) | 106 (57.6) | 63 (54.8) | 33 (78.6) | 341 (78.0) | 30 (83.3) |
| > 1day | 388 (53.2) | 172 (31.5) | 805 (26.5) | 50 (22.7) | 959 (62.7) | 26 (30.9) | 445 (35.0) | 78 (42.4) | 52 (45.2) | 9 (21.4) | 96 (22.0) | 6 (16.7) |
| **Character of stool** | | | | |  |  |  |  |  |  |  |  |
| Non-watery | 22 (3.0) | 4 (0.7) | 15 (0.5) | 1 (0.5) | 77 (5.0) | 4 (4.8) | 33 (2.6) | 4 (2.2) | 2 (1.7) | 0 (0.0) | 0 (0.0) | 0 (0.0) |
| Watery | 707 (97.0) | 542 (99.3) | 3029 (99.5) | 219 (99.6) | 1452 (95.0) | 80 (95.2) | 1237 (97.4) | 180 (97.8) | 113 (98.3) | 42 (100.0) | 437 (100.0) | 36 (100.0) |
| **Presence of blood in stool** | | | | |  |  |  |  |  |  |  |  |
| Absent | 624 (85.6) | 514 (94.1) | 2929 (96.2) | 209 (95.0) | 1171 (76.6) | 73 (86.9) | 1152 (90.7) | 162 (88.0) | 101 (87.8) | 40 (95.2) | 421 (96.3) | 34 (94.4) |
| present | 105 (14.4) | 32 (5.9) | 115 (3.8) | 11 (5.0) | 358 (23.4) | 11 (13.1) | 118 (9.3) | 22 (12.0) | 14 (12.2) | 2 (4.8) | 16 (3.7) | 2 (5.6) |
| **Number of stools in last 24 hours** | | | | |  |  |  |  |  |  |  |  |
| ≤10 times | 283 (38.8) | 193 (35.4) | 790 (25.9) | 41 (18.6) | 792 (51.8) | 34 (40.5) | 408 (32.1) | 60 (32.6) | 47 (40.9) | 23 (54.8) | 87 (19.9) | 7 (19.4) |
| >10 times | 446 (61.2) | 353 (64.6) | 2254 (74.1) | 179 (81.4) | 737 (48.2) | 50 (59.5) | 862 (67.9) | 124 (67.4) | 68 (59.1) | 19 (45.2) | 350 (80.1) | 29 (80.6) |
| **Vomiting in the last 24 hours** | | | | |  |  |  |  |  |  |  |  |
| No | 101 (13.8) | 26 (4.8) | 308 (10.1) | 36 (16.4) | 423 (27.7) | 13 (15.5) | 329 (25.9) | 57 (31.0) | 11 (9.6) | 2 (4.8) | 42 (9.6) | 4 (11.1) |
| Yes | 628 (86.2) | 520 (95.2) | 2736 (89.9) | 184 (83.6) | 1106 (72.3) | 71 (84.5) | 941 (74.1) | 127 (69.0) | 104 (90.4) | 40 (95.2) | 395 (90.4) | 32 (88.9) |
| **Abdominal pain** | | | | |  |  |  |  |  |  |  |  |
| No | 475 (65.2) | 255 (46.7) | 1410 (46.3) | 111 (50.5) | 951 (62.2) | 31 (36.9) | 428 (33.7) | 77 (41.9) | 74 (64.3) | 20 (47.6) | 207 (47.4) | 18 (50.0) |
| Yes | 254 (34.8) | 291 (53.3) | 1634 (53.7) | 109 (49.5) | 578 (37.8) | 53 (63.1) | 842 (66.3) | 107 (58.1) | 41 (35.7) | 22 (52.4) | 230 (52.6) | 18 (50.0) |
| **Fever (Temperature >38°C)** | | | | |  |  |  |  |  |  |  |  |
| absent | 709 (97.3) | 538 (98.5) | 3002 (98.6) | 213 (96.8) | 1463 (95.7) | 80 (95.2) | 1235 (97.2) | 179 (97.3) | 107 (93.0) | 41 (97.6) | 431 (98.6) | 35 (97.2) |
| present | 20 (2.7) | 8 (1.5) | 42 (1.4) | 7 (3.2) | 66 (4.3) | 4 (4.8) | 35 (2.8) | 5 (2.7) | 8 (7.0) | 1 (2.4) | 6 (1.4) | 1 (2.8) |
| **Assessment of dehydration** | | | | |  |  |  |  |  |  |  |  |
| No dehydration | 198 (27.2) | 12 (2.2) | 87 (2.9) | 6 (2.7) | 1000 (65.4) | 14 (16.7) | 123 (9.7) | 20 (10.9) | 31 (27.0) | 2 (4.8) | 11 (2.5) | 3 (8.3) |
| Some/severe dehydration | 531 (72.8) | 534 (97.8) | 2957 (97.1) | 214 (97.3) | 529 (34.6) | 70 (83.3) | 1147 (90.3) | 164 (89.1) | 84 (73.0) | 40 (95.2) | 426 (97.5) | 33 (91.7) |
| **Rehydration method used in hospital** | | | | |  |  |  |  |  |  |  |  |
| Oral rehydration solution (ORS) | 387 (53.1) | 86 (15.7) | 387 (12.7) | 25 (11.4) | 1403 (91.8) | 44 (52.4) | 519 (40.9) | 96 (52.2) | 60 (52.2) | 7 (16.7) | 48 (11.0) | 2 (5.6) |
| Intravenous (IV) fluid | 342 (46.9) | 460 (84.3) | 2657 (87.3) | 195 (88.6) | 126 (8.2) | 40 (47.6) | 751 (59.1) | 88 (47.8) | 55 (47.8) | 35 (83.3) | 389 (89.0) | 34 (94.4) |
| **Length of hospital stay** | | | | |  |  |  |  |  |  |  |  |
| <24 hours | 439 (61.0) | 396 (73.9) | 2159 (71.7) | 117 (53.7) | 1168 (76.9) | 73 (89.0) | 1052 (83.9) | 139 (75.5) | 68 (60.7) | 34 (80.9) | 321 (73.8) | 15 (41.7) |
| ≥24 hours | 281 (39.0) | 140 (26.1) | 850 (28.3) | 101 (46.3) | 351 (23.1) | 9 (11.0) | 202 (16.1) | 45 (24.5) | 44 (39.3) | 8 (19.1) | 114 (26.2) | 21 (58.3) |
