# Supplementary material for: Epidemiology and clinical profile of diarrhea associated with enterotoxigenic Escherichia coli and Vibrio cholerae in Bangladesh: Findings from a hospital-based surveillance system, 2008–2023
Source: PLoS Negl Trop Dis. 2025 Nov 7;19(11):e0013697. doi: 10.1371/journal.pntd.0013697 (PMC12611153; doi:10.1371/journal.pntd.0013697)
Supplement: S2 Table — (DOCX) [file pntd.0013697.s002.docx]

| **Clinical characteristics** | ***V. cholerae*** | | | | **ETEC** | | | | **Co-Infection** | | | |
| --- | --- | --- | --- | --- | --- | --- | --- | --- | --- | --- | --- | --- |
|  | **2008-23**  **aOR**  **(95%CI)**  **p-value** | **2008-12**  **aOR**  **(95%CI)**  **p-value** | **2013-17**  **aOR**  **(95%CI)**  **p-value** | **2018-23**  **aOR**  **(95%CI)**  **p-value** | **2008-23**  **aOR**  **(95%CI)**  **p-value** | **2008-12**  **aOR**  **(95%CI)**  **p-value** | **2013-17**  **aOR**  **(95%CI)**  **p-value** | **2018-23**  **aOR**  **(95%CI)**  **p-value** | **2008-23**  **aOR**  **(95%CI)**  **p-value** | **2008-12**  **aOR**  **(95%CI)**  **p-value** | **2013-17**  **aOR**  **(95%CI)**  **p-value** | **2018-23**  **aOR**  **(95%CI)**  **p-value** |
| **Duration of diarrhea preceding arrival at hospital (>1 Day)** | 0.53  (0.50, 0.57)  **<0.001** | 0.58  (0.52, 0.65)  **<0.001** | 0.46  (0.40, 0.53)  **<0.001** | 0.53  (0.47, 0.60)  **<0.001** | 0.81  (0.75, 0.88)  **<0.001** | 0.98  (0.85, 1.12)  0.766 | 0.84  (0.72, 0.98)  **0.024** | 0.67  (0.59, 0.76)  **<0.001** | 0.41  (0.34, 0.50)  **<0.001** | 0.51  (0.37, 0.71)  **<0.001** | 0.37  (0.24, 0.57)  **<0.001** | 0.38  (0.29, 0.51)  **<0.001** |
| **Watery stool** | 4.35  (3.18, 5.95)  <**0.001** | 5.92  (3.37, 10.37)  **<0.001** | 2.80  (1.86, 4.22)  **<0.001** | 7.92  (2.92, 21.52)  **<0.001** | 1.26  (1.04, 1.53)  **0.0**16 | 1.23  (0.88, 1.72)  0.224 | 1.07  (0.81, 1.40)  0.648 | 1.41  (0.87, 2.29)  0.164 | 12.33  (3.07, 49.51)  **<0.001** | - | 3.63  (0.89, 14.81)  **0.072** | **-** |
| **Mucus/blood mixed stool** | 0.36  (0.32, 0.41)  **<0.001** | 0.38  (0.30, 0.48)  **<0.001** | 0.40  (0.32, 0.51)  **<0.001** | 0.39  (0.31, 0.48)  **<0.001** | 0.86  (0.78, 0.96)  **0.005** | 0.92  (0.76, 1.12)  0.394 | 0.87  (0.72, 1.05)  0.148 | 0.95  (0.82, 1.11)  0.546 | 0.35  (0.25, 0.50)  **<0.001** | 0.18  (0.07, 0.50)  **0.001** | 0.41  (0.21, 0.81)  **0.011** | 0.44  (0.28, 0.70)  **<0.001** |
| **Number of stools in last 24 hours (>10 times)** | 1.31  (1.22, 1.41)  **<0.001** | 1.28  (1.15, 1.43)  **<0.001** | 1.17  (1.01, 1.36)  **0.033** | 1.87  (1.61, 2.17)  **<0.001** | 0.83  (0.77, 0.90)  **<0.001** | 0.99  (0.87, 1.12)  0.868 | 0.83  (0.71, 0.97)  **0.021** | 0.76  (0.67, 0.87)  **<0.001** | 1.50  (1.25, 1.80)  **<0.001** | 1.30  (0.96, 1.76)  0.091 | 1.08  (0.72, 0.1.63)  0.714 | 1.73  (1.28, 2.35)  **<0.001** |
| **Episodes of vomiting in the last 24 hours** | 2.80  (2.53, 3.10)  **<0.001** | 2.92  (2.50, 3.41)  **<0.001** | 2.74  (2.24, 3.35)  **<0.001** | 2.77  (2.31, 3.31)  **<0.001** | 0.96  (0.88, 1.05**)**  0.375 | 0.96  (0.83, 1.10)  0.515 | 0.99  (0.83, 1.17)  0.899 | 0.99  (0.86, 1.14)  0.864 | 3.16  (2.41, 4.14)  **<0.001** | 3.55  (2.17, 5.78)  **<0.001** | 5.74  (2.51, 13.10)  **<0.001** | 2.41  (1.68, 3.43)  **<0.001** |
| **Abdominal pain** | 0.63  (0.59, 0.67)  **<0.001** | 0.68  (0.61, 0.76)  **<0.001** | 0.75  (0.66, 0.86)  **<0.001** | 0.65  (0.58, 0.73)  **<0.001** | 0.92  (0.85, 0.99)  **0.025** | 0.98  (0.86, 1.13)  0.825 | 1.08  (0.92, 1.26)  0.350 | 0.96  (0.85, 1.08)  0.490 | 0.62  (0.53, 0.73)  **<0.001** | 0.70  (0.51, 0.96)  **0.025** | 0.67  (0.45, 0.99)  **0.045** | 0.62  (0.49, 0.78)  **<0.001** |
| **Fever (Temperature >38°C)** | 0.45  (0.36, 0.58)  **<0.001** | 0.53  (0.40, 0.70)  **<0.001** | 0.28  (0.15, 0.52)  **<0.001** | 0.21  (0.09, 0.47)  **<0.001** | 0.91  (0.74, 1.11)  0.345 | 0.89  (0.69, 1.17)  0.413 | 0.97  (0.66, 1.44)  0.896 | 0.68  (0.41, 1.13)  0.136 | 0.71  (0.43, 1.18)  0.189 | 0.71  (0.35, 1.45)  0.349 | 0.73  (0.23, 2.33)  0.599 | 0.88  (0.36, 2.16)  0.776 |
| **Dehydration** | 5.64  (4.94, 6.43)  **<0.001** | 5.03  (4.14, 6.11)  **<0.001** | 5.77  (4.33, 7.69)  **<0.001** | 6.28  (4.99, 7.92)  **<0.001** | 1.23  (1.12, 1.35)  **<0.001** | 1.34  (1.15, 1.56)  **<0.001** | 1.29  (1.07, 1.56)  **0.009** | 1.07  (0.91, 1.26)  0.422 | 5.29  (3.80, 7.36)  **<0.001** | 5.44  (3.16, 9.38)  **<0.001** | 3.79  (1.77, 8.11)  **0.001** | 6.15  (3.72, 10.18)  **<0.001** |
| **Intravenous rehydration method used** | 7.24  (6.61, 7.94)  **<0.001** | 6.91  (6.03, 7.93)  **<0.001** | 8.90  (7.36, 10.76)  **<0.001** | 6.69  (5.67, 7.89)  **<0.001** | 1.15  (1.04, 1.27)  **0.004** | 1.14  (0.97, 1.34)  0.113 | 1.13  (0.94, 1.37)  0.203 | 1.15  (0.98, 1.34)  0.096 | 8.70  (6.84, 11.06)  **<0.001** | 7.94  (5.36, 11.76)  **<0.001** | 11.31  (6.23, 20.53)  **<0.001** | 7.64  (5.33, 10.96)  **<0.001** |
| **Length of hospital stay ≥24 hours** | 1.81  (1.68, 1.94)  **<0.001** | 1.76  (1.57, 1.98)  **<0.001** | 1.70  (1.47, 1.95)  **<0.001** | 1.91  (1.66, 2.19)  **<0.001** | 0.99  (0.90, 1.09)  0.828 | 1.01  (0.87, 1.16)  0.914 | 0.94  (0.79, 1.12)  0.496 | 0.92  (0.77, 1.11)  0.395 | 1.76  (1.47, 2.09)  **<0.001** | 1.86  (1.36, 2.53)  **<0.001** | 2.32  (1.58, 3.41)  **<0.001** | 1.72  (1.32, 2.26)  **<0.001** |

**S2 Table:** Association of Clinical Characteristics of Patients with Diarrheal Illnesses due to V. cholerae, ETEC and co-Infection (2008-2023)

**Adjusted for age, sex, area of residence, household asset index, use of rehydration solution before arrival, and use of antibiotics before hospitalization. In multiple logistic regression analysis, separate models were run for each outcome given in the first column. A total of thirty models (individual logistic regression) were performed for the final analysis.*

*Outcome variables: Each clinical characteristic is considered as a dependent variable: Duration of diarrhea preceding arrival at hospital [≤1 day (reference), >1 Day], watery stool [no (reference), yes], blood mixed stool [no (reference), yes], number of stools in last 24 hours [≤10 times (reference), >10 times], vomiting in last 24 hours [no (reference), yes], abdominal pain [no (reference), yes], fever [no (reference), yes], some or severe dehydration [no (reference), yes], intravenous rehydration method used [no (reference), yes], length of hospital stay [<24 hours (reference), ≥24 hours].*

*Exposure variables: pathogens [V. cholerae (ref= no V. Cholerae), ETEC (ref= no ETEC), co-infection with both cholera and ETEC (ref=No V. cholerae or ETEC)]*
