# Supplementary material for: Epidemiology and clinical profile of diarrhea associated with enterotoxigenic Escherichia coli and Vibrio cholerae in Bangladesh: Findings from a hospital-based surveillance system, 2008–2023
Source: PLoS Negl Trop Dis. 2025 Nov 7;19(11):e0013697. doi: 10.1371/journal.pntd.0013697 (PMC12611153; doi:10.1371/journal.pntd.0013697)
Supplement: Attachment — Submitted filename: Response to Reviewers.docx [file pntd.0013697.s004.docx]

PNTD-D-25-00854
Epidemiology and clinical profile of diarrhea associated with enterotoxigenic Escherichia coli and Vibrio cholerae in Bangladesh: findings from a hospital-based surveillance system, 2008-2023
PLOS Neglected Tropical Diseases

Dear Dr. Das,

Thank you for submitting your manuscript to PLOS Neglected Tropical Diseases. After careful consideration, we feel that it has merit but does not fully meet PLOS Neglected Tropical Diseases's publication criteria as it currently stands. Therefore, we invite you to submit a revised version of the manuscript that addresses the points raised during the review process.

Please submit your revised manuscript within 60 days Oct 15 2025 11:59PM. If you will need more time than this to complete your revisions, please reply to this message or contact the journal office at [plosntds@plos.org](mailto:plosntds@plos.org). When you're ready to submit your revision, log on to <https://www.editorialmanager.com/pntd/> and select the 'Submissions Needing Revision' folder to locate your manuscript file.

Please include the following items when submitting your revised manuscript:

* A rebuttal letter that responds to each point raised by the editor and reviewer(s). You should upload this letter as a separate file labeled 'Response to Reviewers'. This file does not need to include responses to any formatting updates and technical items listed in the 'Journal Requirements' section below.
* A marked-up copy of your manuscript that highlights changes made to the original version. You should upload this as a separate file labeled 'Revised Manuscript with Track Changes'.
* An unmarked version of your revised paper without tracked changes. You should upload this as a separate file labeled 'Manuscript'.

If you would like to make changes to your financial disclosure, competing interests statement, or data availability statement, please make these updates within the submission form at the time of resubmission. Guidelines for resubmitting your figure files are available below the reviewer comments at the end of this letter.

We look forward to receiving your revised manuscript.

Kind regards,

Vangelis Economou
Guest Editor
PLOS Neglected Tropical Diseases

Elsio Wunder Jr
Section Editor
PLOS Neglected Tropical Diseases

Shaden Kamhawi
co-Editor-in-Chief
PLOS Neglected Tropical Diseases
orcid.org/0000-0003-4304-636XX

Paul Brindley
co-Editor-in-Chief
PLOS Neglected Tropical Diseases
orcid.org/0000-0003-1765-0002

**Additional Editor Comments (if provided):**

**Journal Requirements:**

If the reviewer comments include a recommendation to cite specific previously published works, please review and evaluate these publications to determine whether they are relevant and should be cited. There is no requirement to cite these works unless the editor has indicated otherwise.

[Note: HTML markup is below. Please do not edit.]

**Response:** Dear editor, we would like to express our sincere thanks and gratitude to the reviewers and members of the editorial board for reviewing our manuscript and providing insightful comments on our work. We appreciate the time and effort you and the reviewers have invested in the review process for the PLOS Neglected Tropical Diseases.

We would like to confirm that we have carefully considered the reviewers’ as well as the editor’s comments and suggestions. We are submitting the revised manuscript along with a detailed point-by-point response addressing each of the reviewers' comments. All changes made to the manuscript have been clearly marked as suggested. We look forward to your further feedback and hope the revisions meet the expectations of the journal.

**Reviewers' Comments:**

Reviewer's Responses to Questions

**Key Review Criteria Required for Acceptance?**

As you describe the new analyses required for acceptance, please consider the following:

**Methods**

-Are the objectives of the study clearly articulated with a clear testable hypothesis stated?

-Is the study design appropriate to address the stated objectives?

-Is the population clearly described and appropriate for the hypothesis being tested?

-Is the sample size sufficient to ensure adequate power to address the hypothesis being tested?

-Were correct statistical analysis used to support conclusions?

-Are there concerns about ethical or regulatory requirements being met?

Reviewer #1: Overall, the methods are well-explained and rely on a rigorously collected dataset at icddr,b. I do not have statistical expertise but the analysis seems reasonable.

- Why was the time period 2008-2023 chosen?
- Were multiple comparisons taken into account during the logistic regression analysis for setting the p-value threshold for statistical significance?

**Response:** Dear reviewer, thank you for your careful review and positive feedback.

-Dear reviewer, thank you for your query. We selected the time period 2008–2023 because data on enterotoxigenic *Escherichia coli* (ETEC) were not available prior to 2008. We have included a sentence in the main document to enhance the reader’s understanding (Page: 8, Line: 166-168).

Revised sentence: *The 2008–2023 period was chosen based on our objectives, as it marks the earliest availability of ETEC data.*

-Dear reviewer, thank you for the helpful comment regarding multiple comparisons. To reduce the risk of Type I error associated with conducting multiple logistic regression analyses, we applied the ‘Sidak correction’ to adjust the threshold for statistical significance. We have added this clarification to the statistical methods section of the revised manuscript (Page: 9, Line: 200-204).

Revised sentence: *P-values <0.05 (α) were considered significant for a single hypothesis test. However, we needed to control for the family wise alpha inflation to interpret logistic regression findings since we analyzed ten outcomes that were not independent. ‘Sidak correction’ was used to control the family wise error rate [1]. The new level of significance was calculated*, *α_new_= [1– (1 – α_old_) ^1/n^ ] = [1 – (1 – 0.05) ^1/10^ ] = 0.005.*

Reviewer #2: -Are the objectives of the study clearly articulated with a clear testable hypothesis stated? YES
-Is the study design appropriate to address the stated objectives? YES
-Is the population clearly described and appropriate for the hypothesis being tested? YES
-Is the sample size sufficient to ensure adequate power to address the hypothesis being tested? YES
-Were correct statistical analysis used to support conclusions? YES
-Are there concerns about ethical or regulatory requirements being met? YES

**Response:** Dear reviewer, thank you for your careful review and positive feedback.

Reviewer #3: 1.The inclusion of parental education levels (both maternal and paternal) as covariates in regression models is not sufficiently justified in the manuscript. The theoretical or empirical relevance of these variables to disease occurrence, infection type, or clinical severity is unclear. In contrast, more clinically informative variables—such as duration of illness, presence of severe complications, ICU admission, or mortality—could provide a more comprehensive picture of disease burden and care needs.
2.The age stratification of children is rather coarse, primarily using 5 years as a cut-off. However, previous studies have shown that diarrheal etiology and clinical presentation can vary significantly across finer pediatric age groups (e.g., <6 months, 6–23 months, 24–59 months). It is recommended that the authors adopt a more granular age classification for children to improve the specificity and interpretability of the findings.

**Response:**

1. Dear reviewer, thank you for your careful review. We included maternal and paternal education as covariates because parental education is a well-established social determinant of child health in LMICs. It influences care-seeking behavior, health literacy, household practices, and ultimately susceptibility to infectious diseases. Adjusting for education helps account for socioeconomic and behavioral confounding, thereby strengthening the validity of our models. Therefore, we included parental education level in the regression model.

Thank you for your insightful comment. We agree that variables such as duration of illness, presence of severe complications, ICU admission, and mortality would provide a more comprehensive understanding of the disease burden and care needs. However, unfortunately, such detailed clinical information was not available in our dataset. We have acknowledged this as a limitation in our manuscript (page: 18, line: 383-384).

2. Dear reviewer, thank the reviewer for this valuable suggestion. While we acknowledge that diarrheal etiology may differ across finer pediatric age groups, our surveillance study encompassed a broader population, not restricted to under-5 children. As demonstrated in our earlier cholera work [2], we found that the <5 vs. ≥5 years classification provided an effective balance between epidemiologic relevance and statistical robustness in this context. However, we are planning to do another paper on the under 5 diarrhea.

**Results**

-Does the analysis presented match the analysis plan?

-Are the results clearly and completely presented?

-Are the figures (Tables, Images) of sufficient quality for clarity?

Reviewer #1: The results presented are clear and organized in a logical manner.

**Response:** Dear reviewer, thank you for your positive feedback.

Reviewer #2: -Does the analysis presented match the analysis plan? YES
-Are the results clearly and completely presented? YES
-Are the figures (Tables, Images) of sufficient quality for clarity? THEY ARE CLEAR WITH SOME EDITS SUGGESTED BELOW

**Response:** Dear reviewer, thank you for your valuable feedback.

Reviewer #3: Regarding the seasonal variation (Figure 5): the peak months for ETEC, V. cholerae, and co-infection appear inconsistent across years, with particularly notable spikes in 2023. The authors are encouraged to further elaborate on potential factors contributing to these year-to-year differences, such as abnormal weather patterns, outbreak events, changes in diagnostic protocols, or public health interventions.

**Response:** Dear reviewer, thank you for your careful review. Due to the unavailability of high-resolution climate data and detailed information on potential outbreak events or changes in public health interventions during the study period, we were unable to explore these contributing factors in the current analysis. We agree that including such variables could provide valuable context and improve the interpretation of seasonal trends. We have acknowledged this as a limitation in the revised manuscript (page:18-19, line: 387-389)

Revised sentence*: The absence of high-resolution climate data and detailed records of outbreak events or interventions limited our ability to assess their impact on year-to-year differences during the study period.*

**Conclusions**

-Are the conclusions supported by the data presented?

-Are the limitations of analysis clearly described?

-Do the authors discuss how these data can be helpful to advance our understanding of the topic under study?

-Is public health relevance addressed?

Reviewer #1: In general the conclusions are supported and the authors are careful not to overinterpret their data, which is ultimately correlative.

- An important limitation that was not discussed was the potential contribution of subclinical infections, especially for ETEC. Related is the point that pathogen load information is not available, further limiting interpretations.
- Is the study also limited by a lack of information on cholera vaccination status of patients?
- How surprising is the finding that V. cholerae associated symptoms tend to "dominate" when there is co-infection?

**Response:** Dear reviewer, thank you for your careful review.

- We agree that subclinical infections, particularly with ETEC, are common in endemic settings. The absence of quantitative data on pathogen load limits our ability to distinguish between asymptomatic carriage and clinically relevant infections. We have now included this point in the limitations section of the manuscript and acknowledge that future studies incorporating pathogen load measurements and subclinical infection data would strengthen the interpretation of findings (page: 19, line: 389-391).

Revised sentence: *The absence of quantitative data on pathogen load also limited our ability to distinguish between asymptomatic carriage and clinically significant infections.*

-Yes. This was a limitation of the study. Cholera vaccination has not yet been implemented across all districts of the country. The study period we selected was from 2008 to 2023. In the earlier years, vaccination was not part of a routine program. However, in the later phase, vaccination was carried out among vulnerable populations. Unfortunately, we do not have individual-level vaccination data in our surveillance records. We have included it in our limitation section (page:19, line: 391-393).

Revised sentence: *Information on the cholera vaccination status of patients was not available in the dataset, which restricts our ability to assess the potential impact of prior vaccination on susceptibility, disease severity, or pathogen*.

The finding that *V. cholerae* associated symptoms tend to dominate in cases of co-infection is not entirely surprising, given the well-documented severity and rapid onset of cholera symptoms compared to ETEC infections. However, the quantification and confirmation of this clinical dominance in a co-infection setting based on such a large surveillance dataset is valuable. It highlights the clinical importance of recognizing and prioritizing treatment for *V. cholerae* symptoms even when other enteric pathogens are also present.

Reviewer #2: -Are the conclusions supported by the data presented? YES THEY ARE
-Are the limitations of analysis clearly described? LIMITATIONS HAVE BEEN CLEARLY OUTLINED AND discussed
-Do the authors discuss how these data can be helpful to advance our understanding of the topic under study? YES
-Is public health relevance addressed? YES

**Response:** Dear reviewer, thank you for your positive feedback.

Reviewer #3: This study utilized diarrheal surveillance data from icddr,b hospital in Bangladesh spanning from 2008 to 2023, and analyzed the epidemiological patterns and clinical characteristics of diarrhea cases caused by enterotoxigenic Escherichia coli (ETEC) and Vibrio cholerae.

**Response:** Dear reviewer, thank you for your observation.

**Editorial and Data Presentation Modifications?**

Use this section for editorial suggestions as well as relatively minor modifications of existing data that would enhance clarity. If the only modifications needed are minor and/or editorial, you may wish to recommend “Minor Revision” or “Accept”.

Reviewer #1: - Please doublecheck figure references within the text for accuracy throughout.
- Line 253: "valuable insights" seems a little overblown, since the trend itself is not further interpreted.
- Please consider using a colorblind-friendly color scheme in the figures (especially Figure 3).

**Response:** Dear reviewer, thank you for your valuable suggestions. We have revised our manuscript according to your suggestions (page:15, line:299).

-We have also updated Figure 3 using a colorblind-friendly color scheme. The updated figure is now included in the revised manuscript.

Reviewer #2: -To help readers understand the baseline characteristics, Table 1 provides good demographic characteristics, it would be beneficial to add a column for "Total Patients" or "No Pathogen Identified" to provide a complete picture of the study population from which these groups were drawn, especially since your logistic regression uses "no ETEC or V. cholerae" as a reference.

**Response:** Dear reviewer, thank you for your careful review and valuable suggestions. We have included a column of the total patients in Table 1 and 2 for a better understanding of the reader (Table: 1 and Table: 2).

-The Denominator used should be clear for example, in the results section, "4540(11%) tested positive for V. cholerae, 3070(8%) had ETEC, and 630(1.5%) had co-infection"), it would be helpful to clarify what the denominator was used for percentage (i.e. 43,483 patients, or 8240 patients found to have one of the pathogens?) This is because abstract implies the former, but the results section states "Of these, 8240 were found to have a stool culture positive for ETEC, V. Cholerae, or both pathogens." Clarify this early on

**Response:** Dear reviewer, thank you for your careful review and valuable suggestions. We confirm that the denominator used to calculate the percentages in the results section was the total number of patients included in the analysis (n = 43,483). We have clarified this point in the revised manuscript to avoid any confusion. We have revised our writing in the abstract, providing the denominator for a better understanding of the reader (page: 3, line: 65-66).

-Consider rephasing the sentences, too much numbers and adds ratios can be difficult to read for example you can consider the line in the abstrati to read "Co-infected cases showed significantly higher odds of severe outcomes, including watery stools (aOR: 12.33), high stooling frequency (>10/day, aOR: 1.50), vomiting (aOR: 3.16), and intravenous fluid requirements (aOR: 8.70) compared to single-pathogen infections" making it easy to read

**Response:** Dear reviewer, thank you for your valuable suggestions. We have revised our writing for better understanding of the reader (page: 3, line: 68-70).

-Consistent Terminology: Ensure consistent capitalization and formatting for pathogen names (e.g., V. cholerae vs. V. Cholerae, ETEC). Standard scientific notation uses italics for genus and species. "Intravenous-fluid requirements" (abstract) vs. "intravenous rehydration" (results/discussion). Use one consistent term. "Hospital-stay" vs. "hospitalization" vs. "length of hospital stay."

**Response:** Dear reviewer, thank you for your careful review and valuable suggestions. We have revised our writing and are using consistent terminology throughout the revised manuscript.

Fig 2 and S1 Fig seem to have same information, I would suggest that if you keep them you at least say fig 2 is a summary and the detailed is S1 or just replace fig 2 with S1.

**Response:** Dear reviewer, thank you for your careful review and valuable suggestions. We have replaced Fig 2 with S1 Fig.

Reviewer #3: (No Response)

**Summary and General Comments**

Use this section to provide overall comments, discuss strengths/weaknesses of the study, novelty, significance, general execution and scholarship. You may also include additional comments for the author, including concerns about dual publication, research ethics, or publication ethics. If requesting major revision, please articulate the new experiments that are needed.

Reviewer #1: In this manuscript, Alam and colleagues report a retrospective analysis of a large diarrheal disease dataset from icddr,b focused on clinical profiles and epidemiology of V. cholerae and ETEC co infections, which have been sporadically studied in the past but lacked large scale datasets.

**Response:** Dear reviewer, thank you for your kind consideration.

The study is well-described and the results and conclusions appear robust. I have only minor comments for revision (listed in the different sections) apart from one major concern, which is that a very similar analysis of the same dataset from 2017-2022 was published earlier this year (<https://www.sciencedirect.com/science/article/pii/S1201971224004405>). This study included ETEC-V. cholerae coinfections as part of the analysis, finding that ETEC/V. cholerae co-infection was associated specifically with higher rates of vomiting and dehydration. This is consistent with what the current manuscript reports, which is a useful replication of the analysis, but this goes unaddressed in the submitted work. I do not think that this issue precludes publication, however, the authors must include a description of this study in their revised manuscript as well as a detailed discussion of how their study differs from the previously published work (e.g., timeframe, etc.) and whether the scope of the conclusions differ as well.

**Response:** Dear reviewer, thank you for your valuable suggestions. We have discussed the mentioned manuscript in our revised version of our manuscript (page: 18, line: 380-382).

Revised sentence: *While Amin et al.'s recent study analyzes ETEC infections and co-infections, including those with V. cholerae, using surveillance data from Dhaka from 2017 to 2022, our analysis covers a different time range (2008-2023).*

Reviewer #2: This is a great addition to the understanding of the to pathogens which recent literature has shown that in cholera outbreaks ETEC is also a key pathogen. These data and information from the analysis will go along way to argue for possible combination vaccines.

**Response:** Dear reviewer, thank you for your positive feedback.

Reviewer #3: The current title implies that the study addresses the epidemiology and clinical profile of all diarrheal patients; however, the analysis is limited to those infected with ETEC and V. cholerae. Other common etiologic agents of diarrhea, such as rotavirus and norovirus, were neither addressed nor clarified as excluded. If the study focuses solely on ETEC and V. cholerae, this should be clearly reflected in the title, and the mention of the total number of diarrheal patients (43,483) should be presented with caution to avoid misleading readers.

**Response:** Thank you for your valuable comment. We would like to state that while the total number of diarrheal patients (43,483) is presented, our title explicitly states that the study focuses on diarrhea associated with enterotoxigenic *Escherichia coli* (ETEC) and *Vibrio cholerae*. This wording was chosen deliberately to reflect our focus on these two pathogens, rather than on all causes of diarrhea.

We agree that clarity is important, and to avoid potential confusion, we have mentioned in the text in the Methods and Results sections to make it clear that the analysis is limited to patients who tested positive for ETEC or *V. cholerae*, and that the total number of diarrheal cases is provided only to indicate the size of the surveillance population.

 PLOS authors have the option to publish the peer review history of their article ([what does this mean?](https://track.editorialmanager.com/CL0/https:%2F%2Fjournals.plos.org%2Fplosntds%2Fs%2Feditorial-and-peer-review-process%23loc-peer-review-history/1/010f0198b5236440-ecef9136-b85a-4668-ac1b-28a745ef507d-000000/N0JOqNPjkIucbwRoWuey3oNTuGNslMJd0m5c1UrYPz0=224)). If published, this will include your full peer review and any attached files.


If you choose “no”, your identity will remain anonymous but your review may still be made public.


**Do you want your identity to be public for this peer review?** For information about this choice, including consent withdrawal, please see our [Privacy Policy](https://track.editorialmanager.com/CL0/https:%2F%2Fwww.plos.org%2Fprivacy-policy/1/010f0198b5236440-ecef9136-b85a-4668-ac1b-28a745ef507d-000000/nOmeM09qScBpyxRQUvUQPs89vA4Ax-wz-IiAgPT2lKQ=224).

Reviewer #1: No

Reviewer #2: **Yes:** Michelo Simuyandi

Reviewer #3: No

[NOTE: If reviewer comments were submitted as an attachment file, they will be attached to this email and accessible via the submission site. Please log into your account, locate the manuscript record, and check for the action link "View Attachments". If this link does not appear, there are no attachment files.]

**Figure resubmission:**

While revising your submission, please upload your figure files to the Preflight Analysis and Conversion Engine (PACE) digital diagnostic tool, <https://pacev2.apexcovantage.com/>. PACE helps ensure that figures meet PLOS requirements. To use PACE, you must first register as a user. Registration is free. Then, login and navigate to the UPLOAD tab, where you will find detailed instructions on how to use the tool. If you encounter any issues or have any questions when using PACE, please email PLOS at [figures@plos.org](mailto:figures@plos.org). Please note that Supporting Information files do not need this step. If there are other versions of figure files still present in your submission file inventory at resubmission, please replace them with the PACE-processed versions.

**Response:** Dear Editor, thank you for your valuable suggestions. We have replaced our figures with the PACE-processed versions.

**Reproducibility:**

To enhance the reproducibility of your results, we recommend that authors of applicable studies deposit laboratory protocols in protocols.io, where a protocol can be assigned its own identifier (DOI) such that it can be cited independently in the future. Additionally, PLOS ONE offers an option to publish peer-reviewed clinical study protocols. Read more information on sharing protocols at <https://plos.org/protocols?utm_medium=editorial-email&utm_source=authorletters&utm_campaign=protocols>

*In compliance with data protection regulations, you may request that we remove your personal registration details at any time.* [*(Remove my information/details)*](https://track.editorialmanager.com/CL0/https:%2F%2Fwww.editorialmanager.com%2Fpntd%2Flogin.asp%3Fa=r/1/010f0198b5236440-ecef9136-b85a-4668-ac1b-28a745ef507d-000000/BrrsqbJG-0Snk8iLCz8QFaesKvhC3-iJAgPvdicAgs0=224)*. Please contact the publication office if you have any questions.*

Reference:

1.	Haque MA, Choudhury N, Ahmed ST, Farzana FD, Ali M, Naz F, et al. Enhanced women’s decision-making power after the Suchana intervention in north-eastern Bangladesh: a cluster randomised pre-post study. BMJ open. 2022;12(8):e054148.

2.	Das R, Nasrin S, Palit P, Sobi RA, Sultana A-A, Khan SH, et al. Vibrio cholerae in rural and urban Bangladesh, findings from hospital-based surveillance, 2000–2021. Scientific reports. 2023;13(1):6411.
